# Supplementary figures and images for: Health-related quality of life and associated factors after hip fracture. Results from a six-month prospective cohort study
Source: PeerJ. 2023 Mar 15;11:e14671. doi: 10.7717/peerj.14671 (PMC10024485; doi:10.7717/peerj.14671)

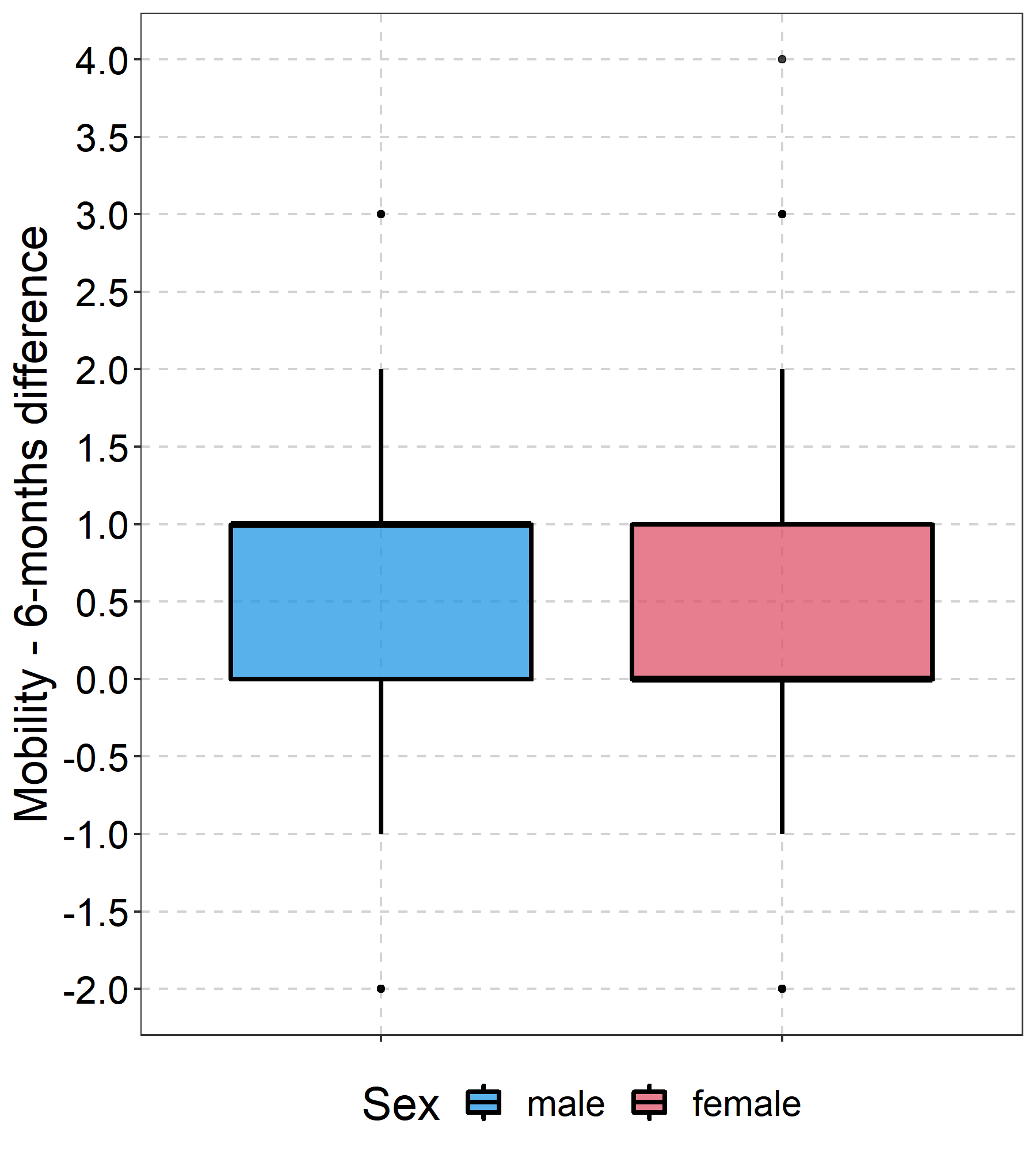

Supplement: Supplemental Information 5 — Boxplot showing the difference between EQ5D Mobility dimension at baseline and follow-up - both for women and men [file peerj-11-14671-s005.png]

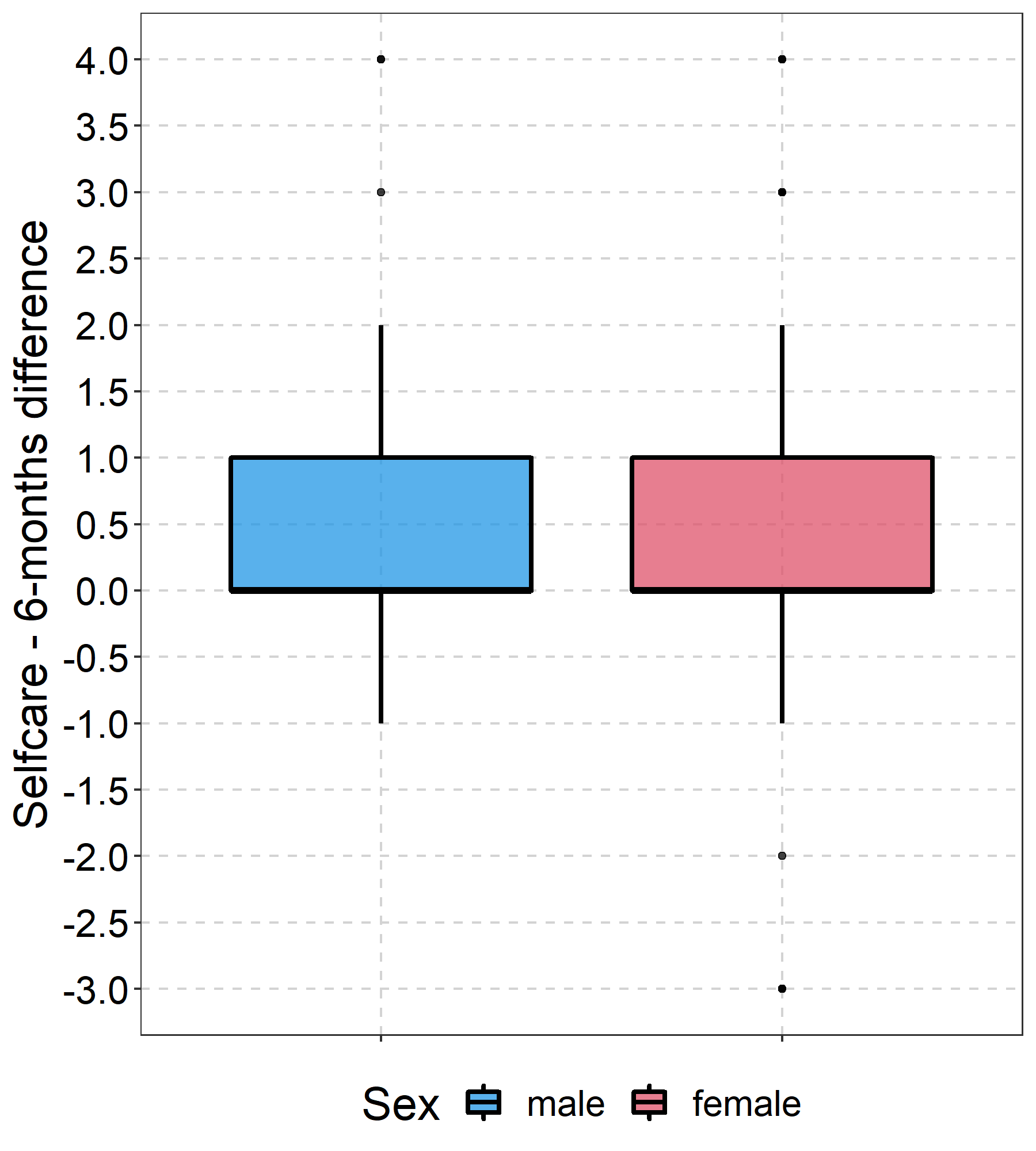

Supplement: Supplemental Information 6 — Boxplot showing the difference between EQ5D Selfcare dimension at baseline and follow-up - both for women and men [file peerj-11-14671-s006.png]

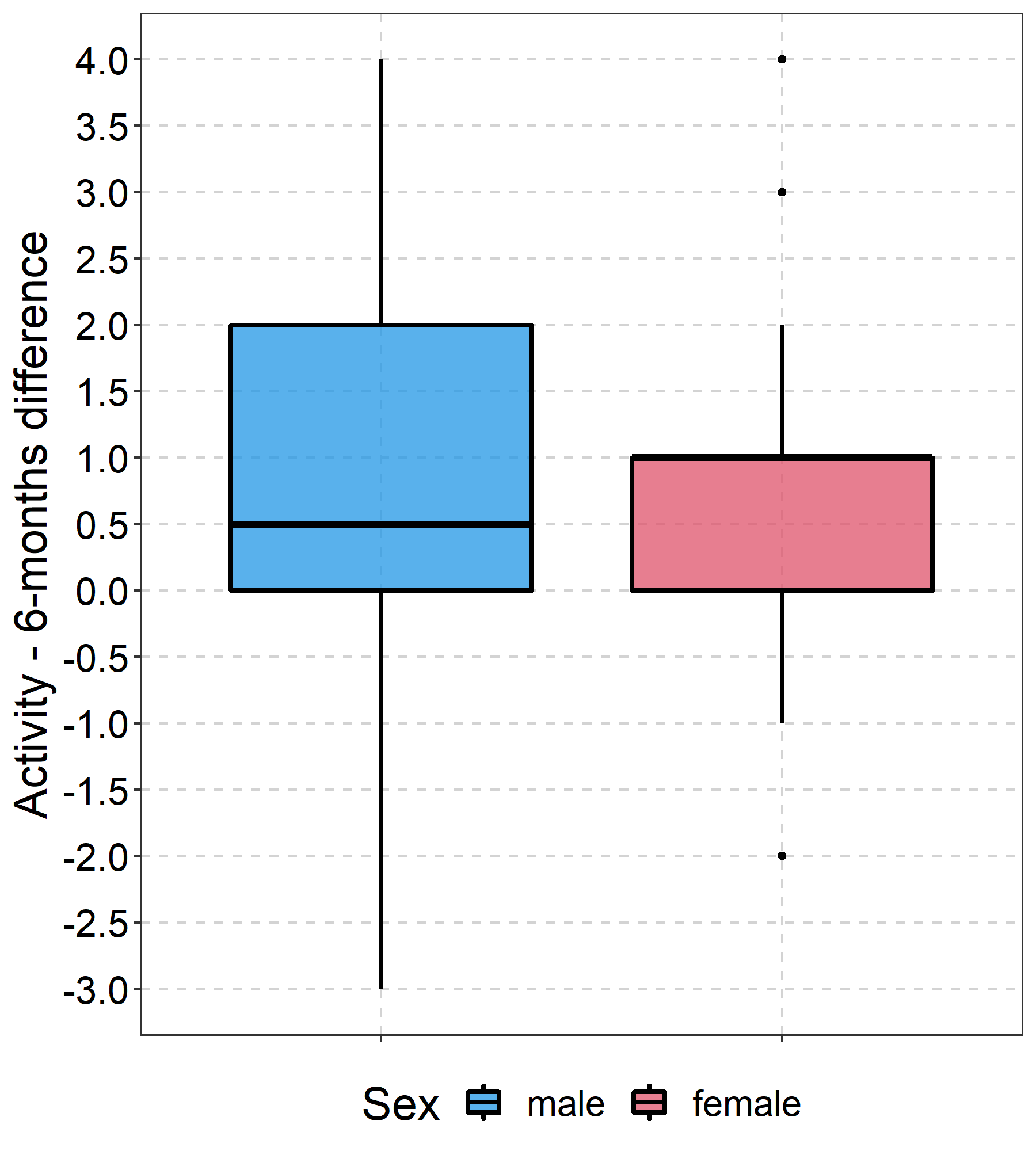

Supplement: Supplemental Information 7 — Boxplot showing the difference between EQ5D Activity dimension at baseline and follow-up - both for women and men [file peerj-11-14671-s007.png]

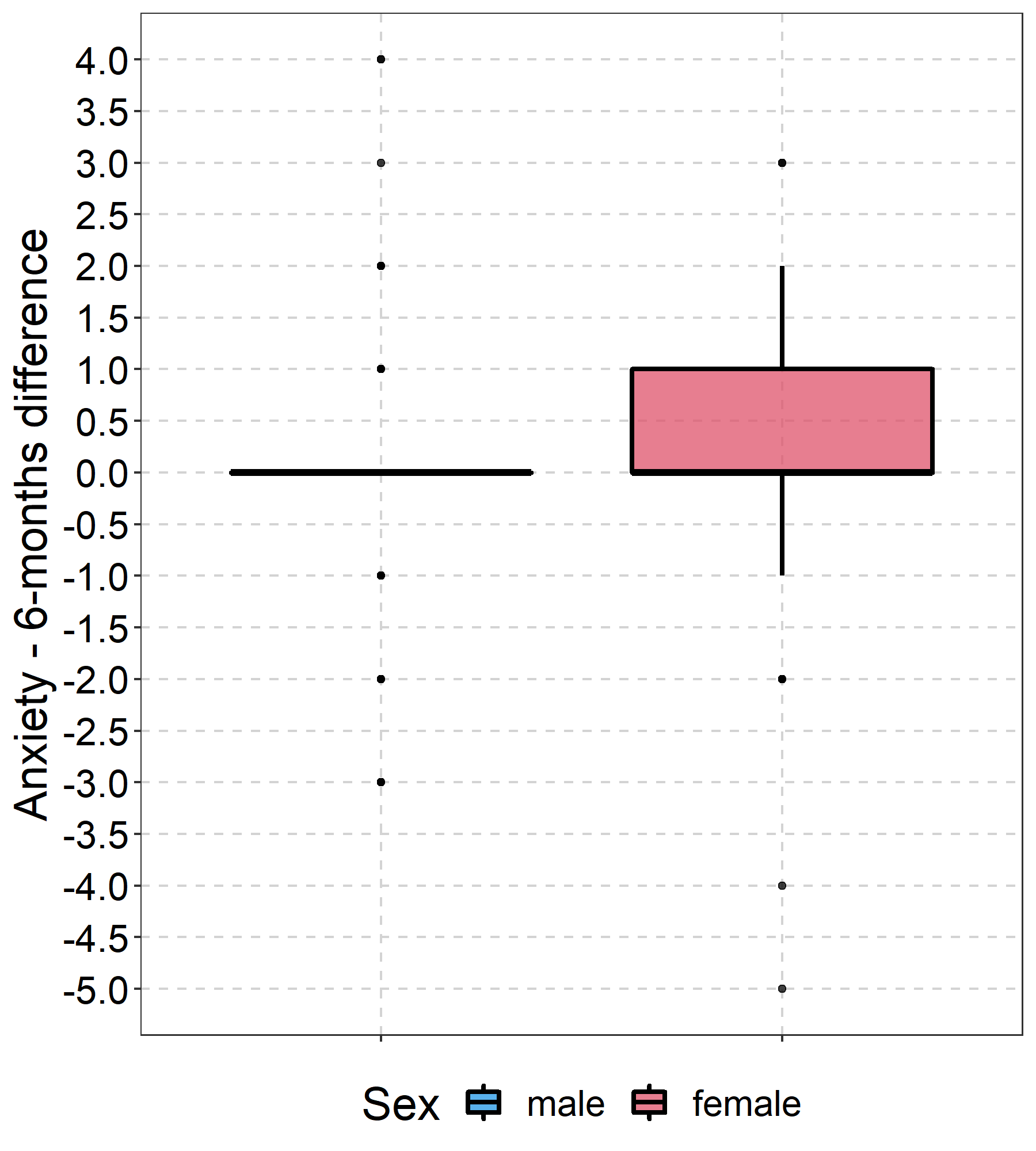

Supplement: Supplemental Information 8 — Boxplot showing the difference between EQ5D Anxiety dimension at baseline and follow-up - both for women and men [file peerj-11-14671-s008.png]

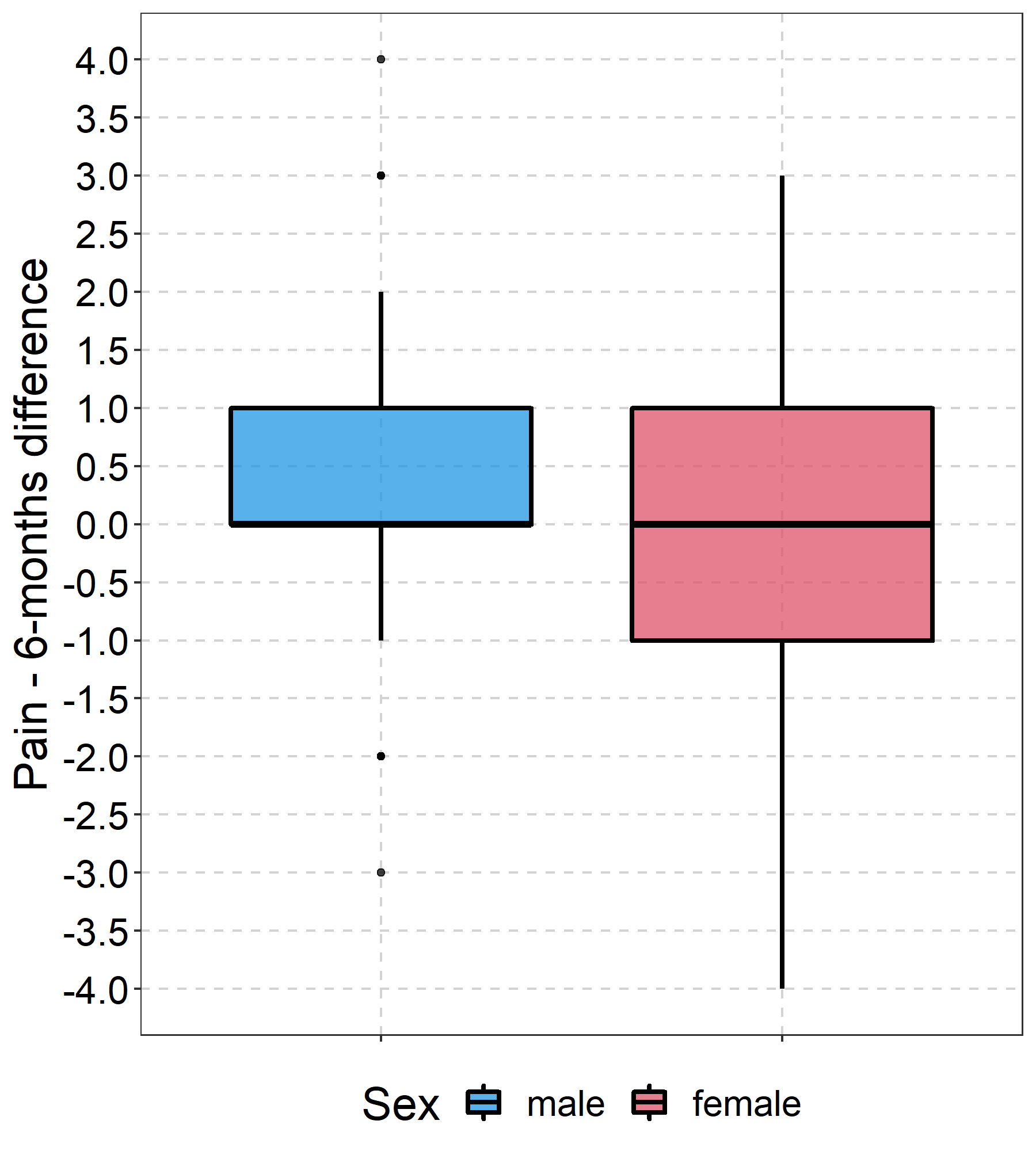

Supplement: Supplemental Information 9 — Boxplot showing the difference between EQ5D Pain dimension at baseline and follow-up - both for women and men [file peerj-11-14671-s009.png]
