# Supplementary material for: Health-related quality of life and associated factors after hip fracture. Results from a six-month prospective cohort study
Source: PeerJ. 2023 Mar 15;11:e14671. doi: 10.7717/peerj.14671 (PMC10024485; doi:10.7717/peerj.14671)
Supplement: Supplemental Information 10 — Sensitivity analysis for the regression model for EQ VAS (Table 3), model including the variable age which was removed in the main model due to non-linear associations between age and the outcome and artificial results [file peerj-11-14671-s010.docx]

**Table 6:** Multivariable linear regression for EQ-VAS at six months – including the variable age

| Predictor | Estimate | SE of regression | Statistic | Degrees of freedom | p-value |
| --- | --- | --- | --- | --- | --- |
| Patient characteristics |  |  |  |  |  |
| Intercept | 10.35 | 17 | 0.61 | 61.9 | 0.545 |
| EQ-VAS baseline value | 0.11 | 0.1 | 1.32 | 41.2 | 0.194 |
| Age | 0.32 | 0.1 | 2.37 | 112 | 0.019 |
| Male sex | 0.8 | 3.1 | 0.26 | 59.3 | 0.797 |
| General hospital | -6.29 | 2.9 | -2.15 | 91.3 | 0.034 |
| Education (reference basic) |  |  |  |  |  |
| Intermediate | -4.53 | 3.7 | -1.23 | 41.2 | 0.226 |
| High | -0.86 | 4.1 | -0.21 | 50.8 | 0.837 |
| Migration | -9.6 | 3.8 | -2.54 | 104.4 | 0.013 |
| Living situation (reference independent with others) |  |  |  |  |  |
| Independent alone | -3.98 | 2.9 | -1.38 | 95 | 0.171 |
| In a facility | -8.72 | 5.1 | -1.71 | 42.1 | 0.095 |
| Proxy | -4.22 | 6.9 | -0.61 | 47.1 | 0.546 |
| Pre-fracture health state & risk factors |  |  |  |  |  |
| Comorbidities (CCI) (reference: 0) |  |  |  |  |  |
| 1 | -4.57 | 4.2 | -1.09 | 49.9 | 0.281 |
| 2 | -4.76 | 4.6 | -1.03 | 54.8 | 0.309 |
| 3+ | -3.94 | 4.6 | -0.85 | 53.5 | 0.398 |
| Pre-fracture dependency | -0.97 | 4.6 | -0.21 | 26.9 | 0.833 |
| Pre-fracture hip functionality (OHS) | 0.71 | 0.3 | 2.16 | 20 | 0.043 |
| Malnutrition | -1.42 | 3.4 | -0.42 | 73.3 | 0.673 |
| Symptoms of depression & anxiety (PHQ-4) | -8.66 | 5.1 | -1.68 | 29.8 | 0.103 |
| Social support: persons to rely on |  |  |  |  |  |
| 3 to 5 | 4.53 | 3.1 | 1.46 | 81 | 0.148 |
| More than 5 | -1.13 | 3.6 | -0.32 | 118.5 | 0.753 |
| Subjective need | -2.05 | 3.1 | -0.66 | 61.1 | 0.513 |
| Polypharmacy | -8.44 | 3.8 | -2.2 | 38.8 | 0.034 |
| Fracture and hospital care |  |  |  |  |  |
| Type of fracture (reference: intracapsular) |  |  |  |  |  |
| Extracapsular | 0.76 | 4.5 | 0.17 | 83.8 | 0.867 |
| Type of surgery (reference: internal fixation) |  |  |  |  |  |
| Arthroplasty | 1.11 | 4.3 | 0.25 | 106.2 | 0.8 |
| ICU episode | -0.05 | 3.2 | -0.02 | 56.2 | 0.988 |
| Referral to a rehabilitation facility | 10.55 | 3.2 | 3.25 | 93 | 0.002 |
| n = 278  R-squared: 0.410, CI [0.315; 0.5]  Adjusted R-squared: 0.351, CI [0.256; 0.445] |  |  |  |  |  |
