# Supplementary material for: Health-related quality of life and associated factors after hip fracture. Results from a six-month prospective cohort study
Source: PeerJ. 2023 Mar 15;11:e14671. doi: 10.7717/peerj.14671 (PMC10024485; doi:10.7717/peerj.14671)
Supplement: Supplemental Information 11 — Sensitivity analysis for the regression model for EQ VAS (Table 3), model including only subjects aged 65 and older [file peerj-11-14671-s011.docx]

**Table 7: Linear regression model for EQ VAS at six months including only subjects 65 years and older**

| Predictor | Estimate | SE of regression | Statistic | Degrees of freedom | p-value |
| --- | --- | --- | --- | --- | --- |
| Patient characteristics |  |  |  |  |  |
| Intercept | 35.56 | 15.6 | 2.29 | 110.2 | 0.024 |
| EQ VAS baseline value | 0.11 | 0.1 | 1.28 | 47.5 | 0.205 |
| Male sex | 0.15 | 3.6 | 0.04 | 80.2 | 0.966 |
| General hospital | -7.43 | 3.5 | -2.1 | 66.3 | 0.039 |
| Education (reference basic) |  |  |  |  |  |
| Intermediate | -4.52 | 3.9 | -1.15 | 65.8 | 0.256 |
| High | 2.72 | 4.9 | 0.55 | 61 | 0.581 |
| Migration | -6.58 | 4.8 | -1.37 | 78.2 | 0.176 |
| Living situation (reference independent with others) |  |  |  |  |  |
| Independent alone | -3.18 | 3.4 | -0.95 | 122.4 | 0.346 |
| In a facility | -12.64 | 6.4 | -1.97 | 26.8 | 0.059 |
| Proxy | -7.63 | 8.6 | -0.88 | 36.7 | 0.383 |
| Pre-fracture health state & risk factors |  |  |  |  |  |
| Comorbidities (CCI) (reference: 0) |  |  |  |  |  |
| 1 | -0.14 | 5.2 | -0.03 | 42.6 | 0.979 |
| 2 | 2.13 | 5.6 | 0.38 | 61.8 | 0.707 |
| 3+ | -1.32 | 5.8 | -0.23 | 41.5 | 0.821 |
| Pre-fracture dependency | -0.32 | 5.1 | -0.06 | 27.7 | 0.951 |
| Pre-fracture hip functionality (OHS) | 0.55 | 0.3 | 1.68 | 44.3 | 0.099 |
| Malnutrition | -0.73 | 4.2 | -0.17 | 45.6 | 0.863 |
| Symptoms of depression & anxiety (PHQ-4) | -5.38 | 5.8 | -0.93 | 38.8 | 0.359 |
| Social support: persons to rely on |  |  |  |  |  |
| 3 to 5 | 2.93 | 4.2 | 0.7 | 34.2 | 0.486 |
| More than 5 | -2.83 | 5.1 | -0.56 | 41.1 | 0.581 |
| Subjective need | -3.8 | 3.7 | -1.02 | 69.7 | 0.313 |
| Polypharmacy | -8.98 | 4.3 | -2.07 | 42.6 | 0.044 |
| Fracture and hospital care |  |  |  |  |  |
| Type of fracture (reference: intracapsular) |  |  |  |  |  |
| Extracapsular | 5.87 | 6.8 | 0.86 | 47.5 | 0.395 |
| Type of surgery (reference: internal fixation) |  |  |  |  |  |
| Arthroplasty | 3.9 | 6.8 | 0.58 | 48.3 | 0.567 |
| ICU episode | -0.46 | 4 | -0.11 | 32.3 | 0.91 |
| Referral to a rehabilitation facility | 10.34 | 3.8 | 2.71 | 89.3 | 0.008 |
| n = 220  R-squared: 0.365, CI [0.259; 0.469]  Adjusted R-squared: 0.287, CI [0.184; 0.395] |  |  |  |  |  |
