# Supplementary material for: Health-related quality of life and associated factors after hip fracture. Results from a six-month prospective cohort study
Source: PeerJ. 2023 Mar 15;11:e14671. doi: 10.7717/peerj.14671 (PMC10024485; doi:10.7717/peerj.14671)
Supplement: Supplemental Information 12 — Sensitivity analysis for the regression model for EQ VAS (Table 3), model including only subjects with complete follow-up data (dropout cases excluded) [file peerj-11-14671-s012.docx]

**Table 8: Linear regression model for EQ VAS at six months including only subjects with full follow-up**

| Predictor | Estimate | SE of regression | Statistic | Degrees of freedom | p-value |
| --- | --- | --- | --- | --- | --- |
| Patient characteristics |  |  |  |  |  |
| Intercept | 34.71 | 15.3 | 2.26 | 79.9 | 0.026 |
| EQ VAS baseline value | 0.12 | 0.1 | 1.43 | 131.5 | 0.156 |
| Male sex | -0.04 | 3 | -0.01 | 177.5 | 0.99 |
| General hospital | -5.59 | 3 | -1.85 | 180.8 | 0.067 |
| Education (reference basic) |  |  |  |  |  |
| Intermediate | -7.09 | 3.4 | -2.07 | 175.5 | 0.04 |
| High | -0.82 | 4 | -0.2 | 167.3 | 0.838 |
| Migration | -8.96 | 4.5 | -2.01 | 175.8 | 0.046 |
| Living situation (reference independent with others) |  |  |  |  |  |
| Independent alone | -3.91 | 3.1 | -1.28 | 179.7 | 0.203 |
| In a facility | -11.35 | 5.4 | -2.11 | 148.1 | 0.036 |
| Proxy | -1.1 | 7.2 | -0.15 | 76 | 0.879 |
| Pre-fracture health state & risk factors |  |  |  |  |  |
| Comorbidities (CCI) (reference: 0) |  |  |  |  |  |
| 1 | -3.51 | 4.2 | -0.85 | 179.6 | 0.399 |
| 2 | -4.19 | 4.6 | -0.91 | 180.7 | 0.365 |
| 3+ | -5.57 | 4.8 | -1.17 | 178.6 | 0.244 |
| Pre-fracture dependency | -0.53 | 4.2 | -0.12 | 162.6 | 0.901 |
| Pre-fracture hip functionality (OHS) | 0.63 | 0.3 | 2.03 | 54.6 | 0.048 |
| Malnutrition | -1.59 | 3.5 | -0.45 | 163.2 | 0.65 |
| Symptoms of depression & anxiety (PHQ-4) | -9.89 | 5 | -1.97 | 131 | 0.051 |
| Social support: persons to rely on |  |  |  |  |  |
| 3 to 5 | 5.16 | 3.3 | 1.56 | 169.3 | 0.119 |
| More than 5 | -0.12 | 3.8 | -0.03 | 179.9 | 0.975 |
| Subjective need | -2.11 | 3.1 | -0.69 | 178.6 | 0.491 |
| Polypharmacy | -6.32 | 3.5 | -1.78 | 162.2 | 0.076 |
| Fracture and hospital care |  |  |  |  |  |
| Type of fracture (reference: intracapsular) |  |  |  |  |  |
| Extracapsular | 3.07 | 4.8 | 0.64 | 181 | 0.523 |
| Type of surgery (reference: internal fixation) |  |  |  |  |  |
| Arthroplasty | 2.59 | 4.6 | 0.56 | 178.5 | 0.575 |
| ICU episode | 1.5 | 3.2 | 0.47 | 178.4 | 0.64 |
| Referral to a rehabilitation facility | 6.84 | 3.3 | 2.09 | 156.5 | 0.039 |
| n = 211  R-squared: 0.386, CI [0.280; 0.488]  Adjusted R-squared: 0.306, CI [0.203; 0.412] |  |  |  |  |  |
